# scMEGA: single-cell multi-omic enhancer-based gene regulatory network inference

**DOI:** 10.1093/bioadv/vbad003

**Published:** 2023-01-12

**Authors:** Zhijian Li, James S Nagai, Christoph Kuppe, Rafael Kramann, Ivan G Costa

**Affiliations:** Institute for Computational Genomics, Joint Research Center for Computational Biomedicine, RWTH Aachen University Medical School, Aachen 52062, Germany; Institute for Computational Genomics, Joint Research Center for Computational Biomedicine, RWTH Aachen University Medical School, Aachen 52062, Germany; Institute of Experimental Medicine and Systems Biology, RWTH Aachen University, Aachen 52062, Germany; Division of Nephrology and Clinical Immunology, RWTH Aachen University, Aachen 52062, Germany; Institute of Experimental Medicine and Systems Biology, RWTH Aachen University, Aachen 52062, Germany; Division of Nephrology and Clinical Immunology, RWTH Aachen University, Aachen 52062, Germany; Department of Internal Medicine, Nephrology and Transplantation, Erasmus Medical Center, Rotterdam 3042, The Netherlands; Institute for Computational Genomics, Joint Research Center for Computational Biomedicine, RWTH Aachen University Medical School, Aachen 52062, Germany

## Abstract

**Summary:**

The increasing availability of single-cell multi-omics data allows to quantitatively characterize gene regulation. We here describe scMEGA (Single-cell Multiomic Enhancer-based Gene Regulatory Network Inference) that enables an end-to-end analysis of multi-omics data for gene regulatory network inference including modalities integration, trajectory analysis, enhancer-to-promoter association, network analysis and visualization. This enables to study the complex gene regulation mechanisms for dynamic biological processes, such as cellular differentiation and disease-driven cellular remodeling. We provide a case study on gene regulatory networks controlling myofibroblast activation in human myocardial infarction.

**Availability and implementation:**

scMEGA is implemented in R, released under the MIT license and available from https://github.com/CostaLab/scMEGA. Tutorials are available from https://costalab.github.io/scMEGA.

**Supplementary information:**

Supplementary data are available at *Bioinformatics Advances* online.

## 1 Introduction

Single-cell RNA sequencing (scRNA-seq) and ATAC-seq (scATAC-seq) techniques provide an unprecedented opportunity to understand gene regulation at the single-cell level by capturing orthogonal molecular information (Li *et al.*, 2021; Zhu *et al.*, 2020). Applying both assays on the same biological samples generates single-cell multi-omics data, which allow to computationally infer gene regulatory networks (GRNs) for different cellular systems, such as fly brain development (Janssens *et al.*, 2022) and human myocardial infarction (Kuppe *et al.*, 2022).

However, such analysis is usually based on complex bioinformatic pipelines that require different tools for each of the steps, such as Seurat for scRNA-seq analysis and data integration (Stuart *et al.*, 2019), ArchR for scATAC-seq analysis and trajectory inference (Granja *et al.*, 2021), chromVAR for TF activity estimation (Schep *et al.*, 2017) and igraph (Csardi and Nepusz, 2006) for network analysis. Currently, three computational tools (Pando, Fleck *et al.*, 2022; CellOracle Kamimoto *et al.*, 2020; FigR Kartha *et al.*, 2022) are available for GRN inference based on single-cell multi-omics profiles. Pando focuses on the identification of regulatory networks limited to TF-TF interaction and does not provide methods for modality integration or trajectory analysis. CellOracle performs analysis of ATAC-seq and RNA-seq data independently. It cannot, therefore, explore gene expression for the delineation of gene-to-peak enhancer links and does not consider TF activity scores at a single-cell level to select relevant transcription factors. FigR, which includes modules for multimodal data integration, peak-to-gene link prediction and trajectory inference, is one of the most comprehensive methods so far. However, it does not support direct operation on Seurat objects and offers few functionalities to explore information from the GRNs.

**Fig. 1.**
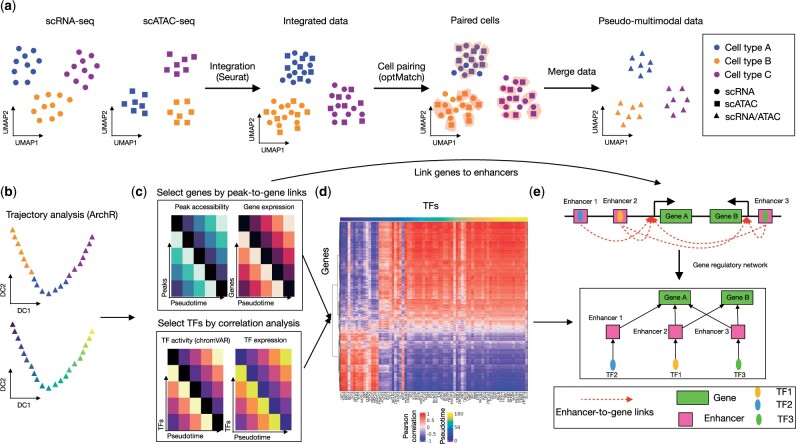
Overview of scMEGA. (**a**) First, scMEGA integrates the single-cell multi-omics profiles to obtain a paired dataset through modality integration built on the R package Seurat and cell pairing built on the approach OptMatch. (**b**) Next, scMEGA infers a pseudotime trajectory to characterize the underlying dynamic process of a given cell type using ArchR. (**c**) It then filters TFs, which are selected based on correlation analysis between binding activity (chromVAR) and expression along the trajectory, and genes, which are selected based on correlation analysis between peak accessibility and gene expression along the trajectory. (**d**) Based on the selected TFs and genes, scMEGA generates a quantitative GRN by estimating the correlation of selected TF and gene expression. (**e**) scMEGA uses enhancer-to-gene links and motif matching to find enhancer-based TF-to-gene interactions. These are used to filter the previously defined quantitative GRN as shown in (d)

We here developed scMEGA as a general framework to quantitatively infer enhancer-based GRN by taking single-cell multi-omics profiles as input. scMEGA, which builds upon expertise on the analysis of multimodal single-cell data of myocardial infarction (Kuppe *et al.*, 2022), enables an end-to-end analysis of multi-omics data for GRN inference including modalities integration, trajectory analysis, enhancer-to-promoter association, network analysis and visualization (Fig. 1). For this, it provides new functionalities and combines some existing methods from Seurat, ArchR, chromVAR and igraph. It is implemented as an R package and is compatible with the Seurat ecosystem.

## 2 Methods

There are three major steps in scMEGA namely, (i) multimodal data integration, (ii) identification and filtering of candidate TFs and genes and (iii) GRN assembly and analysis.

### 2.1 Single-cell multimodal data integration

To build the GRNs, it is crucial to have a mapping between cells at distinct modalities (RNA and ATAC), so that one can find associations between the expression of TF/genes and the activity and accessibility of genes/TFs at the single-cell level. For this, scMEGA first projects the cells into a shared co-embedding space by using the canonical correlation analysis (CCA) implemented by Seurat (Stuart *et al.*, 2019). Here, features are gene expression from scRNA-seq and gene activity scores from scATAC-seq data. In case of batch effects are present, the method Harmony (Korsunsky *et al.*, 2019) can be applied for batch correction. Next, scMEGA performs cell pairing to obtain one-to-one matching between scRNA-seq and scATAC-seq using OptMatch pairing (Kartha *et al.*, 2022). Altogether, these steps build a pseudo-multimodal dataset and a low dimensional representation of the data (Fig. 1a). In the case of single-cell multimodal data with paired cells [e.g. SHARE-seq (Ma *et al.*, 2020) and 10× Multiome], this integration step can be skipped. Here, users only need to create a joint embedding of cells by using for example the CCA integration from MOJITOO (Cheng *et al.*, 2022).

### 2.2 Identification of candidate TFs and genes

scMEGA next identifies candidate TFs and genes based on both multimodal or pseudo-multimodal data from the previous step. First, for the given cells of interest, a pseudotime trajectory characterizing the underlying dynamic process is inferred by using the supervised approach as implemented by the R package ArchR (Granja *et al.*, 2021) (Fig. 1b). Here, the user needs to indicate root and terminal cells, i.e. by their characterization via marker genes.

Then, scMEGA estimates binding activity for each TF in each cell based on chromatin accessibility profiles (Fig. 1c). To identify active TFs, scMEGA calculates the correlation between TF binding activity (estimated with chromVAR; Schep *et al.*, 2017) and TF expression. A high correlation indicates that the TF is both highly expressed and the motif is more accessible than the average profiles (Janssens *et al.*, 2022). This step is crucial, as TF-binding activity alone cannot differentiate between TFs of the same family sharing similar motifs. Next, scMEGA computes the expression variation of each gene along the pseudotime trajectory and picks up the top 10% (this cutoff can be adjusted by the user) most variable genes as trajectory-relevant genes. scMEGA next associates the selected genes to peaks based on the correlation of gene expression and peak accessibility at the single-cell level using functions from ArchR (Granja *et al.*, 2021). Among other features, scMEGA provides functions to allow to directly operate Seurat objects.

### 2.3 GRN construction and visualization

Next, scMEGA builds a quantitative GRN by estimating the correlation of binding activity of all the selected TFs and expression of all the selected genes as described above (Fig. 1d). To link a TF to its target gene, scMEGA first subsets the predicted peak-to-gene links to obtain enhancer-to-gene links where the enhancers are defined as peaks that are at least 2k base pairs (bp) from the transcription start site of a gene (Fig. 1e). It then considers the TF-binding sites as predicted by chromVAR. A gene is only considered the target of a TF if this gene is associated with at least one enhancer and this TF is bound to one of the associated enhancers, which creates an enhancer-based GRN. By combining the quantitative GRN and the enhancer-based GRN, i.e. we only consider TF–gene regulations in both networks, scMEGA produces a final enhancer-based GRN (eGRN). TF–gene interactions are weighted by their correlation.

The directed (from TF to gene) and weighted (as measured by the correlation) GRNs are modeled as a graph by the R package igraph (Csardi and Nepusz, 2006). scMEGA provides eGRN visualization by exploring layout algorithms, such as Fruchterman–Reingold. This layout allows finding major regulatory modules by plotting TFs sharing similar target genes together. Alternatively, users can use the focus layout, which allows for the centralization of the network regarding certain relevant genes/TF. scMEGA also explores network statistics to depict important TFs by computing the page-rank index (Page *et al.*, 1999) or betweenness score (Freeman, 1978) for all TFs or targets. Betweenness scores find regulators (TFs), which bridge distinct modules of the GRN (Zaoli *et al.*, 2021). The page rank detects important TFs, TFs regulating directly or indirectly other genes (Ghoshal and Barabási, 2011). scMEGA allows for visualization of the gene expression of targets of TFs to understand the regulation activity in spatial coordinates.

## 3 Results

### 3.1 Benchmarking the robustness of scMEGA

The integration of multimodal single-cell data is an important step of scMEGA for unpaired single-cell data. To test the impact of this step, we obtained single-cell multimodal data generated by using 10× Multiome protocol from human healthy peripheral blood mononuclear cells. We recovered 10 504 cells and identified fourteen cell types (Supplementary Fig. 1a). Next, we integrated the data and performed cell pairing. We observed that only a few true pairs were correctly recovered (*n* = 41), indicating that cell matching is indeed a difficult task at the single-cell level. However, most of the cells were matched with the correct cell types (71.8%) and mismatching generally represented similar sub-populations, such as non-classical and intermediate monocytes (Supplementary Fig. 1b). These numbers are competitive with a recent benchmarking study (Lance *et al.*, 2022). We next predicted eGRN for CD4 T cells using the true or predicted pairs between scRNA-seq and scATAC-seq data (Supplementary Fig. 1c). Indeed, more than 75% TFs, 83% genes and 60% TF–gene regulations inferred from OptMatch predicted pairs were also supported by the true pairs, indicating that most interactions could be recovered by scMEGA (Supplementary Fig. 1d–f).

### 3.2 Case study on myocardial infarction

We here provide a case study using scMEGA to infer a GRN to study fibrogenesis in human hearts after myocardial infarction (Kuppe *et al.*, 2022). We integrated the snRNA-seq and snATAC-seq data and identified four sub-populations of fibroblasts (Supplementary Fig. 2a). Detection of marker genes indicated that cluster 2 highly expressed *SCARA5* which was recently reported as a marker for myofibroblast progenitors in the human kidney (Kuppe *et al.*, 2021) (Supplementary Fig. 2b). Cluster 1 was marked by *POSTN*, *COL1A1* and *COL3A1*, suggesting that these cells are differentiated myofibroblasts. Based on these, we built a pseudotime trajectory from cluster 2 to cluster 1 to study the myofibroblasts differentiation process (Supplementary Fig. 2c).

**Fig. 2.**
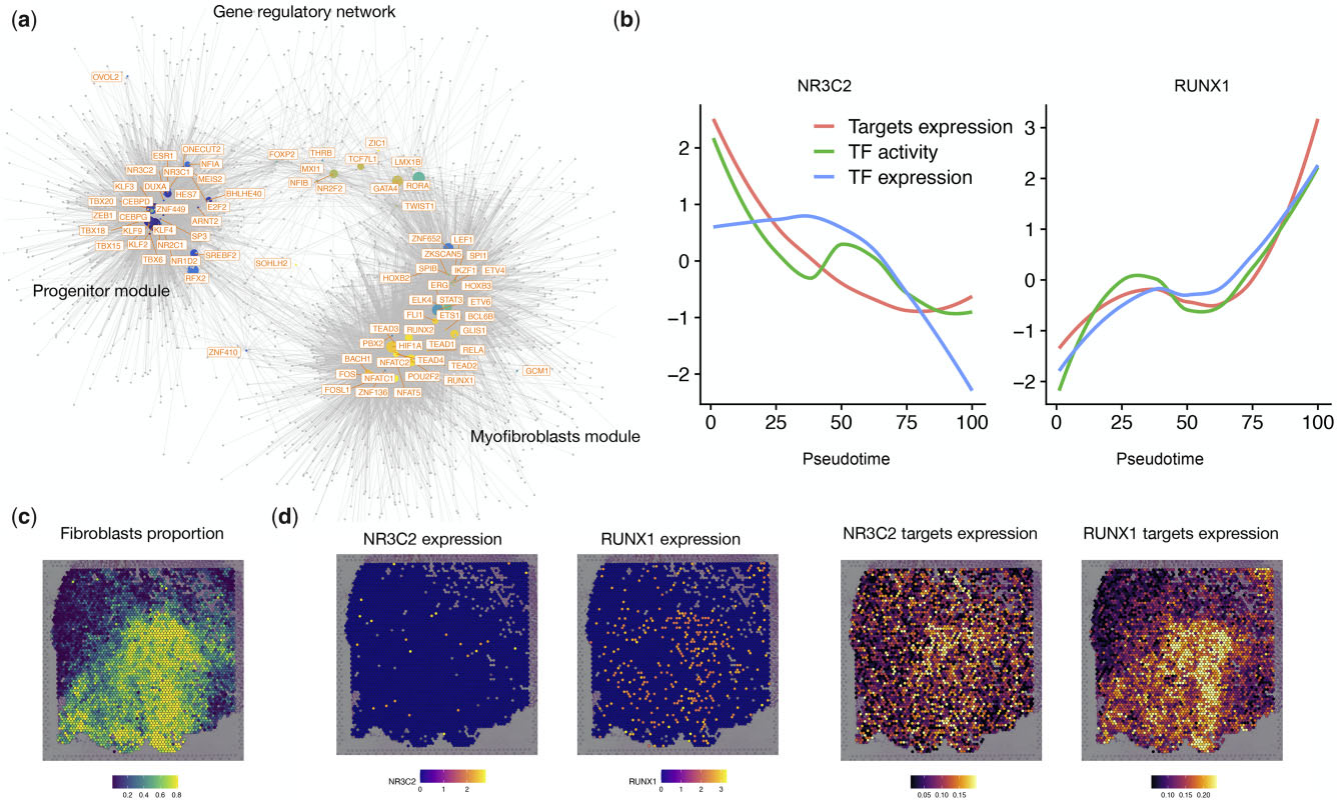
Inferred GRN for myofibroblast differentiation. (**a**) Visualization of the inferred GRN for myofibroblasts differentiation. Each node represents a TF (regulator) or gene (target). TFs are colored by the pseudotime point at which the TF has the highest activity score as estimated by chromVAR. (**b**) Line plots showing the TF binding activity, TF expression and target expression along the myofibroblasts differential trajectory for NR3C2 and RUNX1. The *x*-axis represents the pseudotime points and the y-axis represents the z-score transformed values. (**c**) Visualization of the spatial distribution of fibroblast proportion estimated by cell2location in the ischemic zone of the human heart after myocardial infarction. (**d**) Left: spatial distributed gene expression of NR3C2 and RUNX1. Right: spatially distributed gene expression of all targets of NR3C2 and RUNX1

Next, we selected 79 candidate TFs and 2207 genes as input for inferring GRN (Supplementary Fig. 2d and e). Correlation between the binding activity of the TFs and expression of the genes revealed two major regulation modules with each one corresponding to a distinct fibroblast sub-cluster (Supplementary Fig. 2f). For example, we identified NR3C2 as a regulator of the *SCARA5*+ fibroblasts (module 1—fibroblast progenitors) with a decreased binding activity, TF expression and target gene expression along the trajectory (Fig. 2b). Regarding the myofibroblasts, we detected several fibrosis-relevant TFs as TEAD (Liu *et al.*, 2017) and RUNX family genes. Of note, we have recently characterized the role of RUNX1 as playing an essential role in kidney (Li *et al.*, 2021) and heart (Kuppe *et al.*, 2022) fibrogenesis.

Visualization of the inferred network and regulator properties (between and page-rank scores) pinpointed RUNX1 as the factor with higher importance during myofibroblast differentiation (Supplementary Fig. 3a). A visualization of the eGRN centered around RUNX1 highlights the fact that RUNX1 is predicted to regulate many other genes including other fibrosis-related transcription factors as TEAD2 and TEAD3 (Supplementary Fig. 3b). As another example of downstream analysis allowed by eGRNs, we inspected the expression of target genes of NR3C2 and RUNX1 in space. We could not detect clear expression patterns of the TFs in space due to sparsity and low expression values of these TFs in spatial transcriptomics (Fig. 2c and d). By exploring the regulomes (target genes) of NR3C2 and RUNX1, we observed gradients and mutually exclusive spatial expression in defined cardiac regions of fibrotic responses, highlighting the power of scMEGA in delineating TF regulome expression in sparse spatial transcriptomics data.

## 4 Conclusion

We present scMEGA to infer enhancer-based GRN using single-cell multiomics/multimodal profiles. scMEGA is built upon several R packages for single-cell data analysis. It enables users to perform end-to-end GRN inferences and to prioritize important TFs and genes for experimental validation and the use of regulomes to analyze spatial transcriptomics. We exemplify the use of scMEGA to study gene regulation of myofibroblasts activation in human hearts after myocardial infarction (Kuppe *et al.*, 2022). The data set analyzed here has at least 63 000 and 20 000 cells of snRNA-seq and snATAC-seq, respectively, which demonstrates the scalability of scMEGA. In addition, the data can be generated from different platforms or protocols as batch effects will be corrected computationally using Harmony (Korsunsky *et al.*, 2019). However, the trajectory analysis assumes that cells are part of a differentiation or activation process. Moreover, benchmarking of the single-cell matching problem presented here and by others (Lance *et al.*, 2022) indicates that this is an extremely difficult problem. Future work includes the implementation of additional cell matching algorithms, as top-performing methods reported in (Lance *et al.*, 2022). Altogether, we believe that scMEGA is an important framework for understanding complex gene regulation mechanisms of various biological processes from single-cell multi-omics data.

## Supplementary Material

vbad003_Supplementary_Data

## Data Availability

The data used in this article are available in: https://zenodo.org/record/6623588. Notebooks for the analysis presented in this work are found https://costalab.github.io/scMEGA/articles/myofibroblast-GRN.html.
