# Supplementary material for: scMEGA: single-cell multi-omic enhancer-based gene regulatory network inference
Source: Bioinform Adv. 2023 Jan 12;3(1):vbad003. doi: 10.1093/bioadv/vbad003 (PMC9853317; doi:10.1093/bioadv/vbad003)
Supplement: vbad003_Supplementary_Data [file vbad003_supplementary_data.pdf]

scMEGA: Single-cell Multiomic Enhancer-based Gene Regulatory Network Inference

Zhijian Li[1], James S Nagai[1], Christoph Kuppe[2,3], Rafael Kramann[2,3,4], and Ivan G. Costa[1,*]

[1]Institute for Computational Genomics, Joint Research Center for Computational Biomedicine, RWTH Aachen University Medical School, 52074 Aachen, Germany
[2]Institute of Experimental Medicine and Systems Biology, RWTH Aachen University, Aachen 52062, Germany
[3]Division of Nephrology and Clinical Immunology, RWTH Aachen University, Aachen 52062, Germany
[4]Department of Internal Medicine, Nephrology and Transplantation, Erasmus Medical Center, Rotterdam, The Netherlands
[*]corresponding authors: ivan.costa@rwth-aachen.de

January 10, 2023

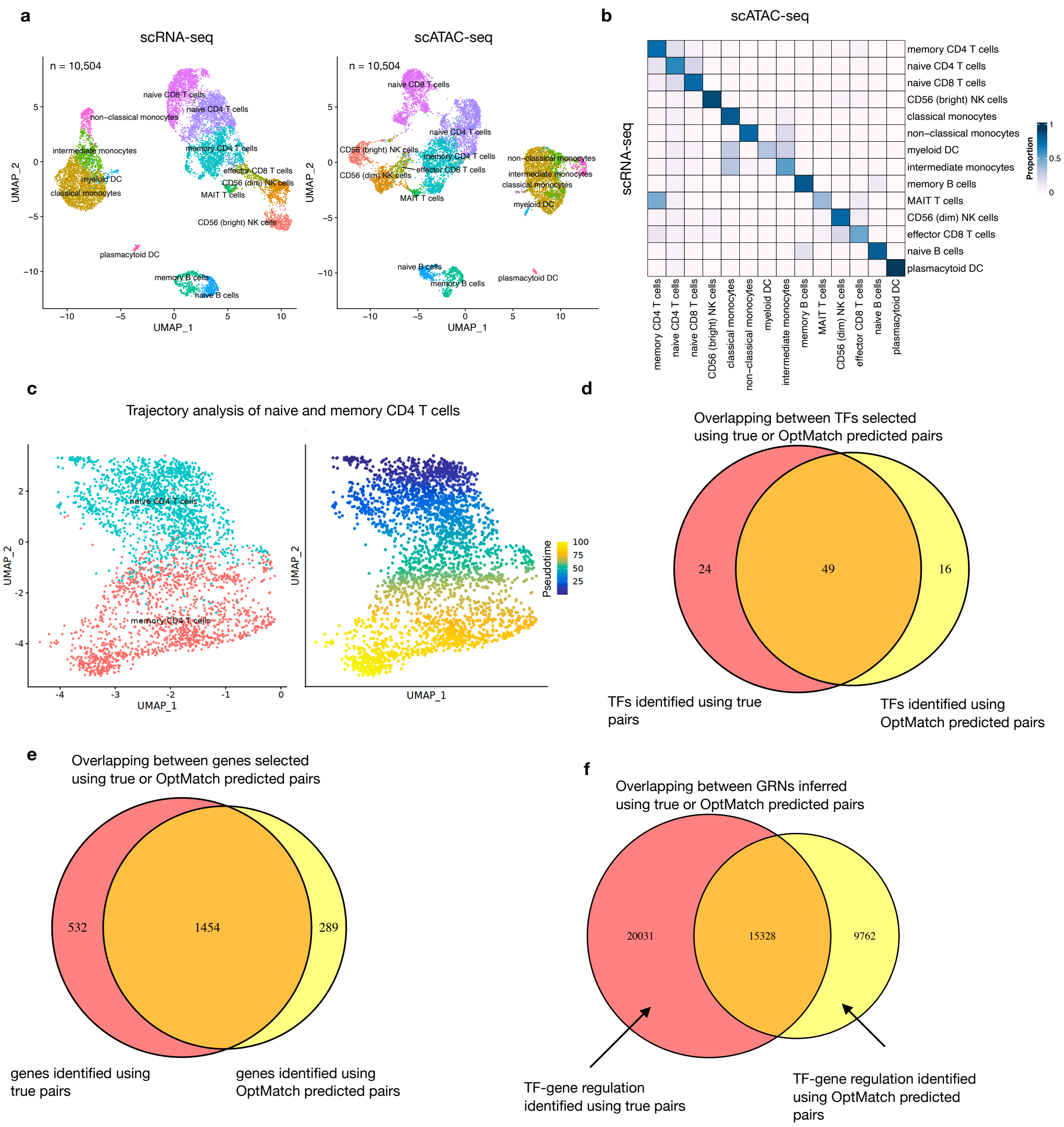

Supplementary Fig. 1. Benchmarking of the robustness of scMEGA using single-cell multimodal PBMC cells. a, UMAP embedding of scRNA-seq (left) and scATAC-seq (right) of PBMC cells. Labels refer to annotated cell types. The number of cells is shown in the left-upper corner. **b,** Confusion matrix showing evaluation results of OptMatch prediction. Colors refer to the proportion of predicted pairs that are from the same cell types. **c,** Trajectory analysis from naive CD4 T cells to memory CD4 T cells characterizing T cells activation. Colors refer to different cell types (left) or pseudotime (right). **d,** Venn diagram showing the overlapping of TFs selected by scMEGA using true or OptMatch predicted pairs between scRNA-seq and scATAC-seq cells. **e,** Same as **d** for selected genes. **f,** Same as **d** for inferred TF-gene regulations.

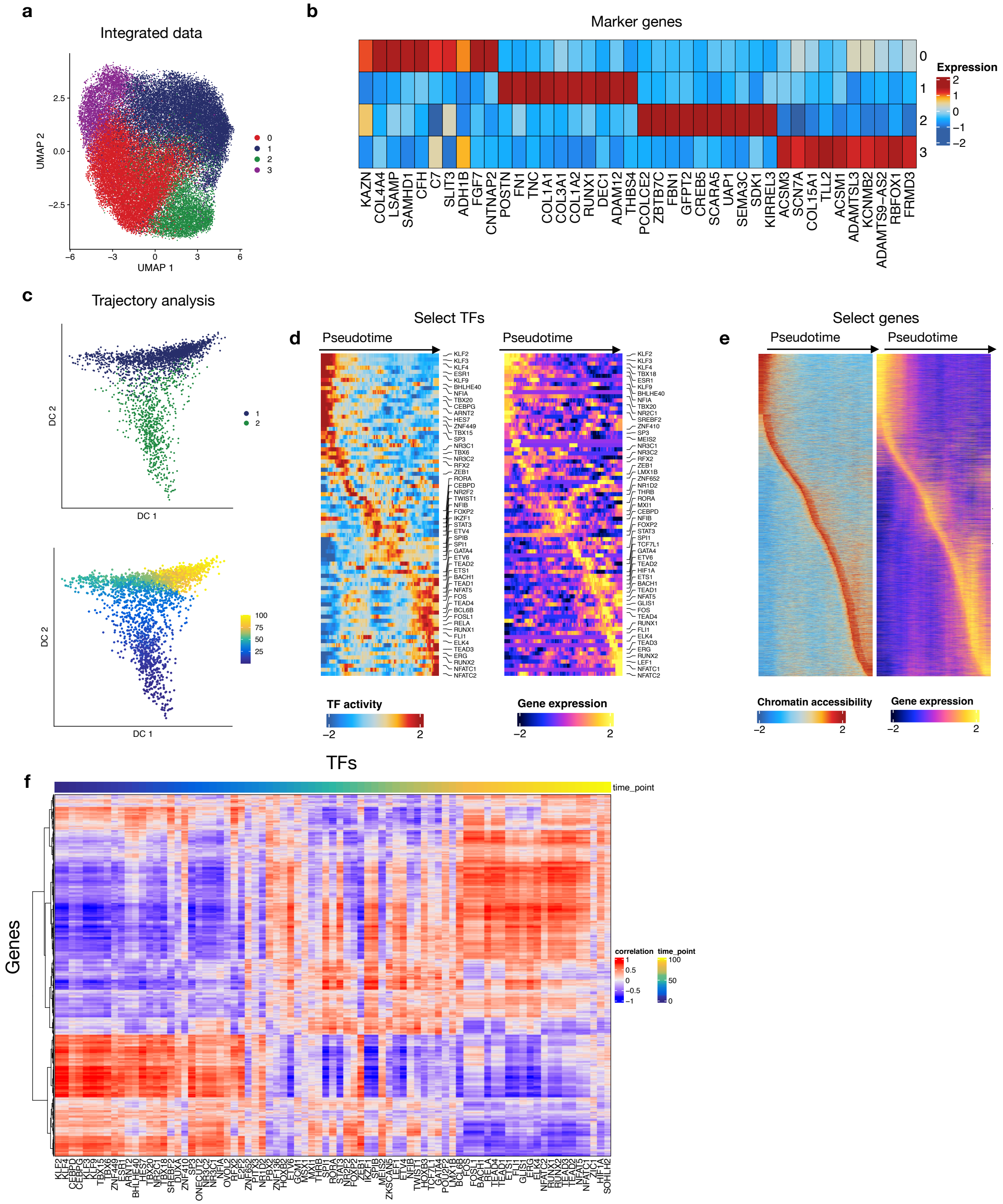

Supplementary Fig. 2. Sub-clustering of integrated snRNA-seq and snATAC-seq for fibroblasts. **a,** UMAP embedding of the integrated data colored by sub-clusters of fibroblasts. **b,** Heatmap showing the expression of the top 10 markers for each cluster after z-score transformation. **c,** Diffusion map showing the differentiation trajectory from the cluster 2 ($SCARA5+$) to the cluster 1 ($POSTN+$) as colored by clusters (upper) and pseudotime points (lower). **d,** Heat maps showing the selected TFs based on the correlation of binding activity and expression along the pseudotime trajectory. **e,** Heat maps showing the selected genes based on the correlation of peak accessibility and gene expression along the pseudotime trajectory. **f,** Heat map of the correlation between the binding activity of the selected TFs and expression of the selected genes.

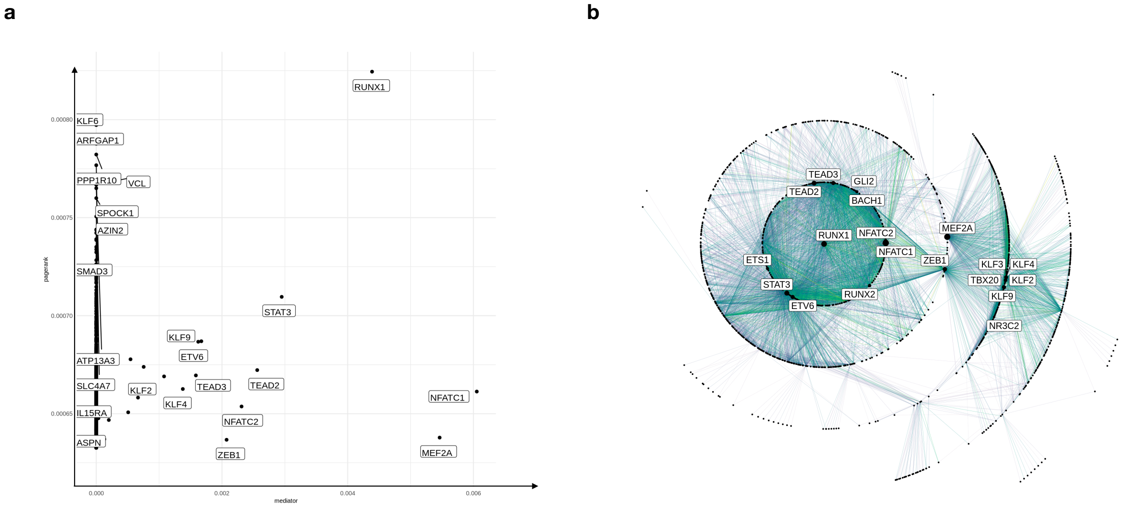

Supplementary Fig. 3. Network analysis identifies important regulators a, Scatter plot showing the topological network measures embedding. The x-axis represents betweenness and the y-axis represents the PageRank scores of regulators. RUNX1 is the only factor, which has high values for both measures. **b,** Runx1-centric layout embedding of the network. It indicates Runx1 has several target genes including other TFs such as Tead3, Tead2 and Bach1.
